# Efficacy and Safety of Non-surgical Versus Surgical Management of Primary Hyperaldosteronism: A Systematic Review and Meta-Analysis

**DOI:** 10.7759/cureus.97427

**Published:** 2025-11-21

**Authors:** Yasin Uddin, Mudassar Khan

**Affiliations:** 1 School of Medicine, Imperial College London, London, GBR

**Keywords:** ablation, embolisation, hypertension, laparoscopic adrenalectomy, minimally invasive, primary aldosteronism

## Abstract

Primary aldosteronism (PA) is an increasingly recognised cause of treatment-resistant secondary hypertension. However, rates of diagnosis and awareness remain low due to under-screening and the challenges of lateralising aldosterone secretion. Adrenal venous sampling (AVS), the gold standard for subtype differentiation, is technically demanding and not widely available, contributing to delayed or suboptimal management. Laparoscopic adrenalectomy (LA) is the current gold-standard treatment for unilateral aldosterone-producing adenomas, but many patients are unfit for or decline surgery. Minimally invasive techniques such as radiofrequency ablation (RFA) and adrenal artery embolisation have emerged as potential alternatives, offering shorter recovery and lower complication risk. This systematic review and meta-analysis evaluated the efficacy and safety of ablative and embolisation therapies compared with LA in the management of PA. PubMed, MEDLINE, Embase, SCOPUS, and Web of Science were systematically searched with no language or year restrictions. Eligible studies directly compared LA with ablation or embolisation in patients with unilateral PA. Data were extracted on clinical and biochemical success (using the Primary Aldosteronism Surgical Outcome (PASO) criteria), complications, operative time, hospital stay, blood loss, hypertensive crises, and antihypertensive medication use. Continuous variables were analysed as mean differences (MD) and categorical outcomes as odds ratios (OR) with 95% confidence intervals (CI) using a Mantel-Haenszel random-effects model. Medians and ranges were converted to means and standard deviations using the Luo/Wan method. Heterogeneity was assessed using I², with sensitivity analyses performed via leave-one-out testing. Eight studies (one prospective, seven retrospective), including 448 patients (193 non-surgical, 255 surgical), were analysed. Clinical success rates were comparable between LA and non-surgical management (90.1% vs. 85.9%; OR: 0.63; 95% CI: 0.40, 1.00; p = 0.05). Biochemical success was also similar (94.7% vs. 95.2%; RR: 0.99; 95% CI: 0.95, 1.04; p = 0.61). Minor complication rates did not differ significantly (OR: 0.73; 95% CI: 0.22, 2.45; p = 0.61), and no major complications were reported in non-surgical cohorts. The mean operative time was significantly shorter in the non-surgical group (MD: −75.28: 95% CI: -126.67, −23.90; p = 0.01). Length of stay, blood loss, and post-procedure serum potassium did not differ significantly between groups. Non-surgical management was associated with a slightly higher number of antihypertensive medications post-procedure (MD: 0.13; 95% CI: 0.01, 0.25; p = 0.04) and a higher, but non-significant, rate of intraoperative hypertensive crises (OR: 1.77; 95% CI: 0.30, 10.46; p = 0.38). Minimally invasive interventions such as ablation and embolisation demonstrate comparable clinical and biochemical efficacy to LA in treating PA, with fewer major complications and reduced operative time. However, they may carry a greater risk of intraoperative hypertensive crises and continued reliance on antihypertensive medication. As global populations age and surgical risk profiles increase, these approaches may offer valuable alternatives, particularly where surgical expertise or AVS capability is limited. Ongoing randomised controlled trials will be crucial in defining their long-term efficacy, safety, and cost-effectiveness.

## Introduction and background

Primary aldosteronism (PA) is a prevalent cause of secondary hypertension, accounting for 5-13% of all cases of hypertension [1-3]. It is associated with an increased risk of cardiovascular and cerebrovascular disease [2-4]. In PA, aldosterone production is increased due to a unilateral aldosterone-producing adenoma (APA) or bilateral adrenal hyperplasia [3]. This leads to an imbalance in the renin-angiotensin-aldosterone system (RAAS), which leads to hypertension and hypokalaemia [3]. Given that hypertension is related to morbidity and mortality worldwide, effective control of blood pressure is vital in preventing end-organ damage and reducing the risk of cardiovascular events [2,5]. Current treatment guidelines recommend a laparoscopic adrenalectomy (LA) for a unilateral PA and mineralocorticoid receptor antagonists (MRA) for bilateral adrenal hyperplasia [6].

Despite the established treatment pathways, the diagnosis of PA remains challenging. Earlier literature reported diagnosis rates as low as 1% in some cohorts of patients with hypertension, largely due to under-recognition and modest screening [7]. Many patients with PA exhibit normokalaemia or mild hypertension, which are often not considered sufficient prompts for further endocrinological work-up, thereby contributing to underdiagnosis [8].

A key challenge in the diagnostic pathway is determining whether aldosterone excess is due to a unilateral APA or bilateral adrenal hyperplasia, because the management differs significantly. Imaging modalities (CT/MRI) may detect adrenal masses but cannot reliably distinguish functional laterality-small adenomas may not be visible, and nonfunctioning adrenal nodules ('incidentalomas') may be present in hypertensive populations [9]. Therefore, lateralisation of aldosterone secretion is essential. For this purpose, adrenal venous sampling (AVS) is considered the gold-standard test for differentiating unilateral from bilateral disease in PA [10,11].

However, AVS is an invasive procedure requiring catheterisation of both adrenal veins, is technically demanding (with variable success rates reported), and demands specialised expertise and infrastructure; it is often available only in select tertiary referral centres [12]. The limited availability of AVS, therefore, contributes to diagnostic delays or incomplete subtype classification. As a result, some patients may receive sub-optimal therapy (e.g., medical rather than surgical when surgery might have yielded a cure) or have protracted follow-up because of an incomplete work-up.

In patients treated with surgery, outcomes are assessed using the Primary Aldosteronism Surgical Outcome (PASO) criteria, which defines different levels of clinical and biochemical successes to determine response [5]. The current recommended treatment for APA is LA due to its benefits in achieving both clinical and biochemical success, namely, improving hypokalaemia and reducing blood pressure, as well as the need for antihypertensive medication [11,13].

Though LA is an effective treatment option, surgery carries with it risks and can prolong hospital stay and recovery time. Non-surgical techniques such as ablation and embolisation offer an alternative for patients who are unable to undergo surgery or for patients for whom surgery or medical treatment is unacceptable. Ablation can be catheter-based or percutaneous and uses imaging techniques such as ultrasound or CT to destroy adrenal tissue [11,14]. Embolisation also uses a catheter-based technique but involves occluding the arterial supply to part or all of the adrenal tissue to reduce or stop its function [15]. Previous literature has reported comparable efficacy of non-surgical techniques in achieving long-term clinical and biochemical successes compared to the gold-standard treatment, as well as improved safety [11,14,15].

To date, limited literature exists to review current studies comparing the efficacy of adrenalectomy against minimally invasive techniques. This systematic review and meta-analysis will pool data from the latest studies to evaluate the efficacy and safety of non-surgical techniques compared to LA.

## Review

Methods

Search Strategy

The protocol for this meta-analysis was designed in adherence with the Preferred Reporting Items for Systematic Reviews and Meta-Analyses (PRISMA) standards [16]. A comprehensive and exhaustive literature search of the following databases was conducted: PubMed, MEDLINE, Embase, Scopus, and Web of Science. Focused keywords were used, including 'Hyperaldosteronism', 'Adrenalectomy', 'Ablation', 'Embolisation', and associated Medical Subject Headings (MeSH) terms. The full details of the search strings used are listed in the Appendices. The results of the search were exported into Covidence (Covidence systematic review software, Veritas Health Innovation, Melbourne, Australia), which automatically removed duplicates.

Eligibility Criteria

Retrieved articles were screened by two independent authors, and any difference in opinion was resolved through discussion. The following inclusion criteria were employed to screen results: (1) studies involving patients with aldosterone-producing tumours; (2) human studies only; (3) completed RCTs and cohort studies (prospective and retrospective); (4) direct comparison between surgical management and either ablative or embolisation methods; and (5) measurement of efficacy and complications. Studies not directly comparing the two interventions and preliminary trial results were excluded, along with reviews, case reports, and letters. No restrictions were applied on language or year of publication due to the novelty and dearth of research on this topic. Following the completion of full-text screening, reference checking of included articles and seminal papers was done to identify any studies which might have been missed from the original search.

Quality Appraisal 

The Newcastle-Ottawa Scale (NOS) was used to assess the quality of the studies included in this meta-analysis [17]. This checklist was chosen as a well-established and validated tool in the assessment of non-randomised cohort studies. Two authors conducted the quality appraisal, scoring each of the included studies across eight domains grouped under three categories: selection, comparability, and outcome. The NOS operates on a star-based scoring system whereby reviewers check if a study has adhered to the highest-ranked standard within each domain. There is a maximum of four stars achievable for each of selection and outcome, and a maximum of two stars available for comparability. By totalling up the number of stars, studies were assigned a quality rating of 'good quality', 'fair quality', or 'poor quality'.

Data Extraction

Both authors evaluated the full texts, figures, tables, and supplementary materials/appendices (if applicable) to extract data. To summarise baseline characteristics, the following data were extracted: study design, publication year, base country, population size, age, sex, BP, serum potassium, and treatment procedure. Outcomes extracted were clinical treatment success, biochemical success, complication rates, operative time, length of hospital stay, resolution of hypokalaemia, blood loss, and number of anti-hypertensive medications. This study utilised the PASO study’s definitions for clinical success and biochemical success [5]. Clinical success is defined by having a normal BP post-procedure without the aid of anti-hypertensive medication or having the same BP as pre-procedure with less anti-hypertensive medication or a reduction in BP post-procedure with either the same or fewer anti-hypertensives. Biochemical success is defined as correction of hypokalaemia (if hypokalaemia was present pre-procedure) and normalisation of the aldosterone-renin ratio (ARR) or a positive aldosterone suppression test. Alternatively, a correction of hypokalaemia with a raised ARR with one or both of the following conditions being met as compared with pre-procedure: ≥50% decrease in plasma aldosterone concentration, or abnormal but improved aldosterone suppression test result. Complication outcomes were stratified as minor or major according to the Clavien-Dindo Classification, with minor complications corresponding to Grades I and II, and major complications corresponding to Grades III and IV [18]. Numerical data were extracted into MS Excel (Microsoft Corporation, Redmond, Washington, United States) and reviewed by both authors to prevent errors. 

Statistical Analysis 

Analysis of numerical data was conducted in Review Manager (RevMan, Version 9.12.2; The Cochrane Collaboration). Continuous variables were expressed as mean difference (MD) alongside 95% confidence intervals (CI). Categorical variables were presented as odds ratios (OR) or risk ratios (RR) with 95% CI. Due to study heterogeneity, the Mantel-Haenszel random effects model was applied to reduce bias when comparing results from across the studies. Whereby studies reporting outcomes as medians with ranges, these variables were converted to estimated means with standard deviations using methods described by Luo and colleagues [19]. The I^2 ^statistic, representing percentage variability between included studies, was used to quantitatively assess heterogeneity. I^2^ is interpreted with the following thresholds: 0% no observed heterogeneity; 25-50% moderate heterogeneity; and >50% high heterogeneity. A result of >50% triggered sensitivity analysis and consideration of switching to a fixed-effect model. The method of sensitivity analysis employed in this study was the leave-one-out approach to assess whether the exclusion of studies one at a time had a meaningful effect on the resultant outcome.

Assessment of Publication Bias

Formal assessment of publication bias was not performed because fewer than ten studies were included in this meta-analysis. Conventional methods for detecting publication bias, such as funnel plots, Egger’s test, and Begg’s test, require a sufficient number of studies to ensure adequate statistical power. Given the limited number of studies, any assessment would be underpowered and potentially unreliable.

Results

Study Characteristics 

**Figure 1:**
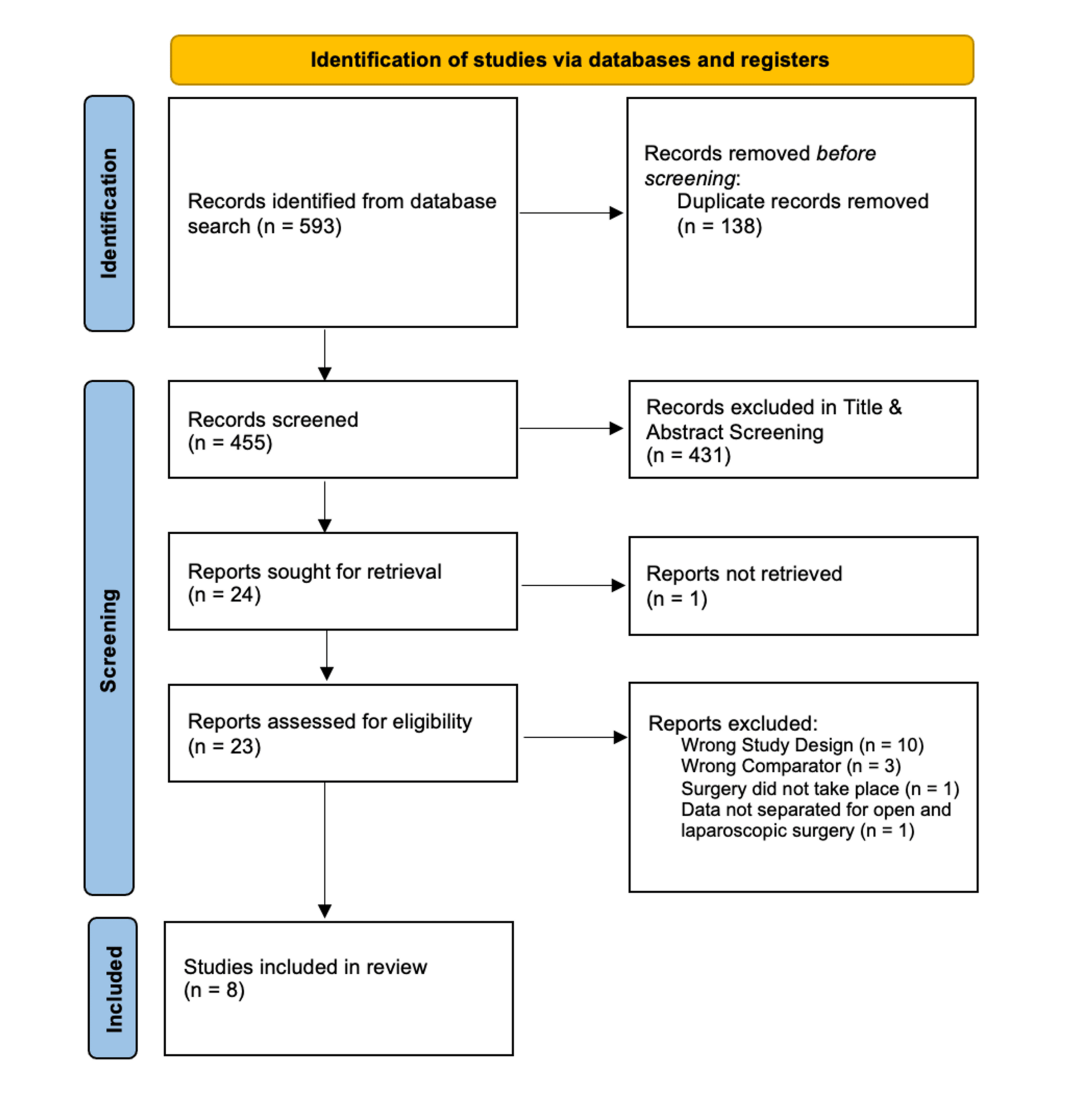
PRISMA flow diagram summarising the screening and text selection process PRISMA: Preferred Reporting Items for Systematic Reviews and Meta-Analyses

Eight studies were included in this systematic review. The full screening process is detailed in the PRISMA diagram in Figure 1. No further articles matching the inclusion criteria were identified from reference checking. The characteristics of the seven studies are listed in Table 1. Trials that exclusively included patients with bilateral adrenal adenomas or that failed to make a distinction between unilateral and bilateral tumours in the presented data were excluded. One study focused on adrenal metastases was excluded as a significant proportion of their surgical cohort underwent open adrenalectomy, and outcomes were not separately reported for laparoscopic and open surgery, respectively. 

**Table 1:** Summary of characteristics of included studies RFA: radiofrequency ablation; NA: not available

Authors	Year of publication	Country	Study design	Total number of participants	Total number of participants relevant to this study	Mean age (years)	Sex ratio (male/female)	Number of participants in non-surgical group	Treatment method	Mean baseline blood pressure (mmHg)	Mean baseline serum potassium (mmol/L)	No. of participants in surgical group	Mean baseline blood pressure (mmHg)	Mean baseline serum potassium (mmol/L)
Sun et al. [14]	2023	China	Prospective	112	112	43.9	0.96	52	Catheter-based adrenal ablation (with ethanol)	147/91	3.29	60	145/89	3.10
Cano-Valderrama et al. [20]	2022	Spain	Retrospective	34	34	55.1	1.83	10	CT-guided RFA	NA	NA	24	NA	NA
Liu et al. [21]	2020	China	Retrospective	60	60	54.9	4.45	29	US-guided RFA	NA	NA	31	NA	NA
Liu et al. [22]	2016	China	Retrosective	63	63	51.6	0.80	36	CT-guided RFA	157/97	2.60	27	144/85	2.70
Sarwar et al. [23]	2016	USA	Retrospective	44	44	50.3	1.10	12	CT-guided RFA	145/94	3.2	32	144/89	3.5
Wang et al. [24]	2025	China	Retrospective	379	72	48.6	0.85	35	Superselective adrenal artery embolisation (with ethanol)	155.60/ 96.46	3.61	37	149.43/ 90.81	3.39
Yang et al. [25]	2016	Taiwan	Retrospective	25	25	NA	1.57	7	CT-guided RFA	153/92	2.30	18	163/98	2.40
Yang et al. [26]	2014	China	Retrospective	38	38	NA	0.09	12	Retroperitoneoscopic-guided cool-tip RFA	3.10	172.5/ 98.3	26	167.3/ 97.1	3.03

The search yielded seven retrospective studies and one prospective study, published between 2014 and 2025 [14,20-26]. Across all articles, there was a total population of 448 patients (193 underwent non-surgical management and 255 were surgically managed). Six of the studies used RFA as their non-surgical intervention. This was guided by ultrasound, CT or retroperitoneoscopically. The remaining methods employed were superselective adrenal artery embolisation (SAAE) and catheter-based adrenal ablation, both with ethanol.

Quality Assessment

Using the NOS, all eight included studies received a rating of 'good quality' with individual domain breakdowns (Table 2). Seven out of eight studies scored the maximum number of stars (nine out of nine). The solitary paper not to score the maximum was the only prospective study by Sun et al. The loss of one star in comparability was because they failed to control for additional variables which could contribute to the cure of PA. At baseline, there was a statistically significant difference in serum potassium and renin levels between the group undergoing surgery and the group undergoing ablation. They also recruited patients in the ablation group who had already failed medical management with magnetic resonance angiography (MRA), suggesting these patients may have a more aggressive or treatment-resistant form of aldosteronism. These factors were not adjusted for or evaluated with multivariate regression.

**Table 2:** Quality assessment of all eight studies using the NOS for cohort studies

Study	Study year	Study design	Selection (maximum 4 stars)	Comparability (maximum 2 stars)	Outcome (maximum 3 stars)	Overall quality (good, fair, poor)
Wang et al. [24]	2025	Retrospective	★★★★	★★	★★★	Good
Sun et al. [14]	2023	Prospective	★★★★	★	★★★	Good
Cano-Valderrama et al. [20]	2022	Retrospective	★★★★	★★	★★★	Good
Liu et al. [21]	2020	Retrospective	★★★★	★★	★★★	Good
Liu et al. [22]	2016	Retrospective	★★★★	★★	★★★	Good
Sarwar et al. [23]	2016	Retrospective	★★★★	★★	★★★	Good
Yang et al. [25]	2016	Retrospective	★★★★	★★	★★★	Good
Yang et al. [26]	2014	Retrospective	★★★★	★★	★★★	Good

Clinical Success

**Figure 2:**
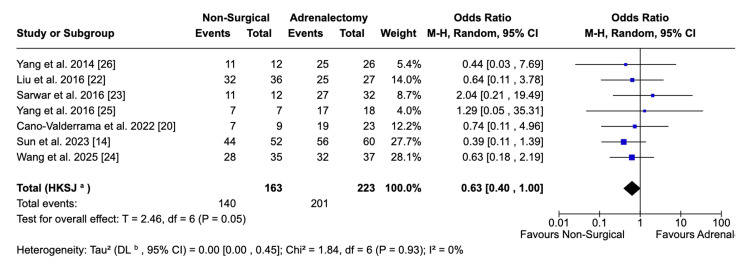
Forest plot visualising comparative clinical success rates between surgical and non-surgical populations

Clinical success rate was similar between surgically and non-surgically managed patients at 90.1% and 85.9%, respectively (OR: 0.63; 95% CI: 0.40, 1.00; p = 0.05). This was pooled from seven studies as showcased in Figure 2. There was no observed heterogeneity in the computing of this outcome (I^2^ = 0%). This supports the premise of this study, suggesting ablative and embolisation therapies are a viable alternative to LA. However, the difference in clinical success rates showed borderline significance.

Biochemical Success

**Figure 3:**
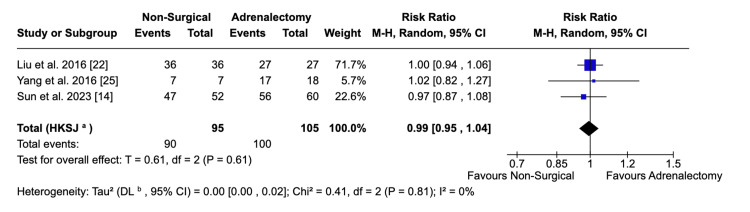
Forest plot visualising comparative biochemical success rates between surgical and non-surgical populations

Across pooled data from three studies, biochemical success was almost identical between non-surgical and surgical management of PA (94.7% and 95.2%, respectively; p = 0.61) (Figure 3). There was no observed heterogeneity in the computing of this outcome (I^2^ = 0%).

Complications

**Figure 4:**
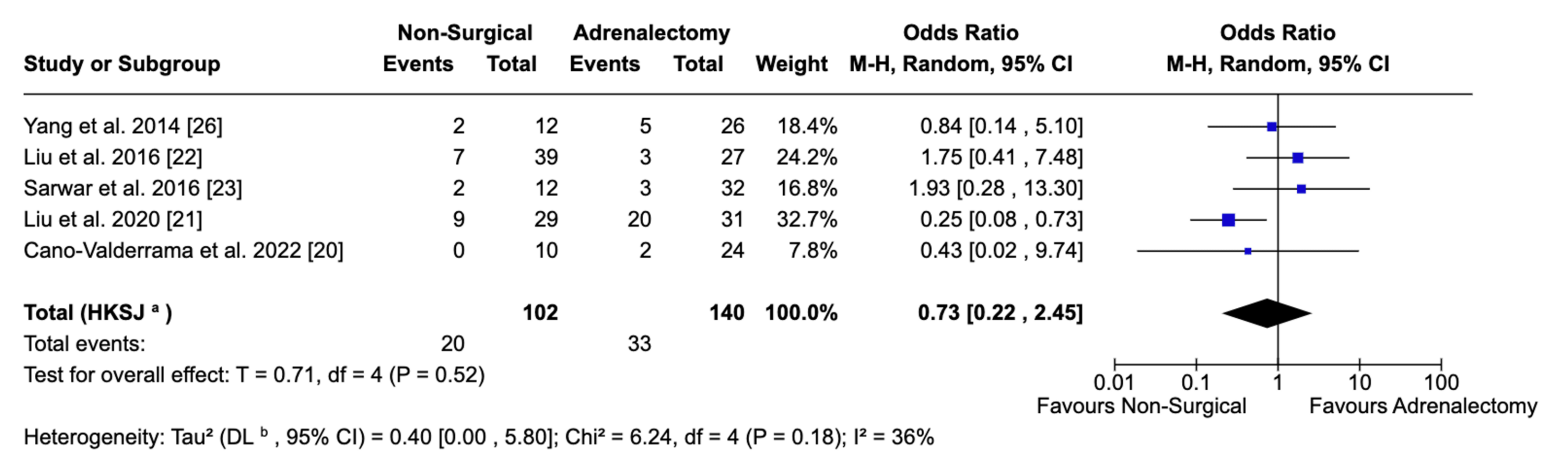
Forest plot visualising the rate of minor complications between surgical and non-surgical populations

The incidence of minor complications, as defined by the Clavien-Dindo system [18], was similar between both groups (OR: 0.73; 95% CI: 0.22, 2.45; p = 0.61). This was derived from data pooled across five studies (Figure 4) with moderate heterogeneity (I^2^ = 36%). Across all studies with complication data, there were zero major complications among patients undergoing ablation or embolisation. Consequently, this data will be described narratively rather than calculating an OR. There were seven incidences of patients suffering major complications from the cohorts of the four studies. 

Operative Time

**Figure 5:**
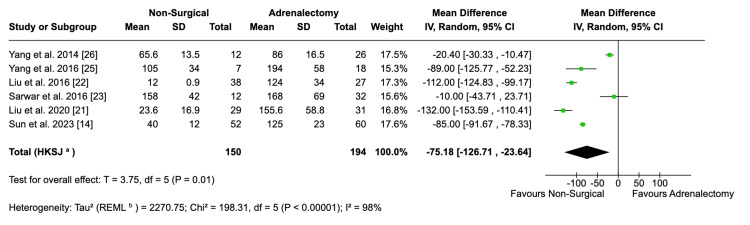
Forest plot visualising comparative operative time between surgical and non-surgical populations

The length of operating time was significantly higher in patients undergoing adrenalectomy compared to ablation/embolisation (MD: -75.28; 95% CI: -126.67; -23.90; p = 0.01). This result was computed from data pooled from six studies (Figure 5). However, there is significant heterogeneity in this result (I^2^ = 98%), which was not remediated by leave-one-out sensitivity analysis. This is likely to be driven by variation in skill level and experience between the doctors undertaking the procedures; differences in equipment quality and availability in different geographies; and a lack of a formal, consistent method of measuring the start and end points of the procedure.

Length of Hospital Stay

**Figure 6:**
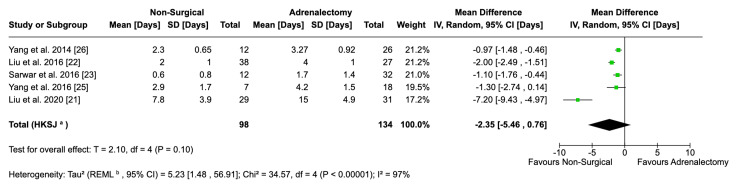
Forest plot visualising comparative hospital stay lengths between surgical and non-surgical populations

Pooled data from five eligible studies did not show a statistically significant difference between the mean length of hospital stay in both types of procedures (MD: -2.35; 95% CI: -5.46, 0.76; p = 0.10) (Figure 6). Notably, this is despite the individual studies all reporting a longer mean hospital stay for their LA patients. This result needs to be viewed in the context of high heterogeneity (I^2^ = 97%). Sensitivity analysis did not show any one study having a disproportionate impact on the results. Therefore, the source of heterogeneity is likely from differences in study design and post-procedure protocol. In future studies, a standardised protocol for discharge and follow-up care should be utilised to garner more comparable data.

Hypertensive Crises 

**Figure 7:**
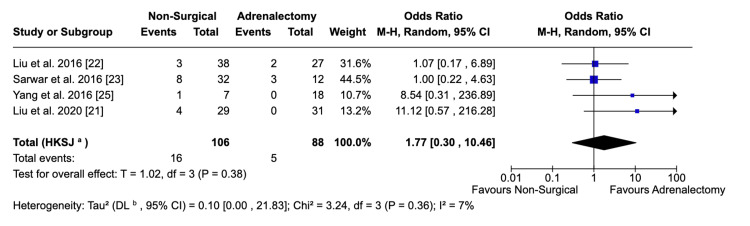
Forest plot visualising comparative incidences of hypertensive crises between surgical and non-surgical populations

Data pooled from four studies identified no statistically significant difference in the rate of hypertensive crises between the two groups (OR: 1.77; 95% CI: 0.30, 10.46; p = 0.38) (Figure 7). Six of the included articles reported this as an outcome, but in two studies, zero patients experienced a crisis, so they did not contribute to the pooled outcome. This result showed a low level of heterogeneity (I^2^ = 7%).

Bloods Loss 

**Figure 8:**
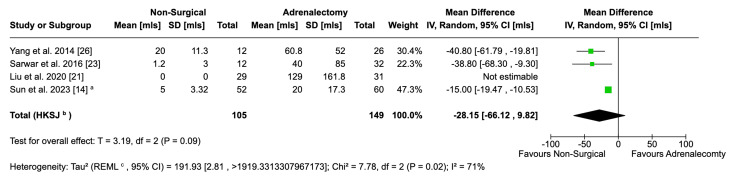
Forest plot visualising comparative volume of blood loss between surgical and non-surgical populations ^a^ Mean and standard deviation data from this study were converted from medians and ranges using the Luo-Wan method

The mean volume of blood loss was not significantly different between the two groups, from data pooled across three studies (MD: -28.15; 95% CI: 66.12, 9.82; p = 0.09). One further study, illustrated in Figure 8, reported data on this metric, but an MD was not estimable. This is because the study in question utilised a percutaneous ablation approach, so it reported no blood loss and no SD. Because of this, Review Manager was not able to calculate the variance. Liu et al. [22] also collected data on blood loss, but this was reported as a percentage change in haemoglobin concentration, so it was not comparable to the volume of blood lost and hence excluded from the meta-analysis. 

The result from three studies shows a high level of heterogeneity (I^2^ = 71%). When excluding the data from Sun et al., this drastically reduced heterogeneity (I^2^ = 0%) [14]. This does not change the directionality of the result, which still identifies greater blood loss from LA. But, it does make the difference statistically significant (p = 0.01). The disproportionate influence of Sun et al. is likely due to differences in study design and ablation technique. They reported blood loss as a median, so the conversation to estimate the mean might also have had an effect. This revised result must be treated with caution as it is computed from data from only two studies. 

Serum Potassium

**Figure 9:**
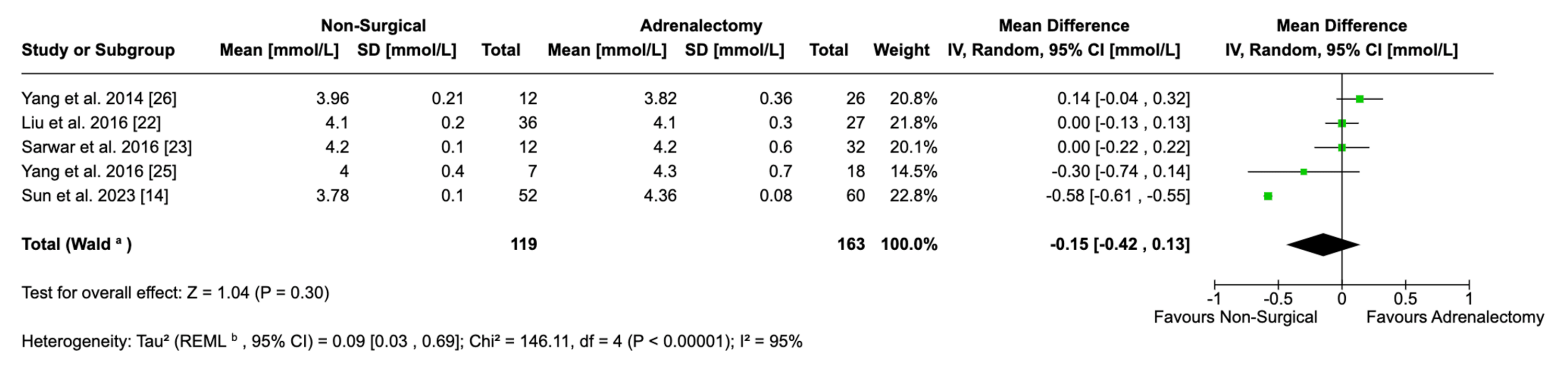
Forest plot visualising comparative serum potassium between surgical and non-surgical populations

Pooled data from five studies showed that there was no significant difference between mean serum potassium levels post-procedure between non-surgically managed and surgically managed patients (MD: -0.15; 95% CI: -0.42, 0.13; p = 0.30) (Figure 9). This result exhibited a high degree of heterogeneity (I^2^ = 95%). Removing the data from Sun et al. in the sensitivity analysis completely alleviated this (I^2^ = 0%) and had a negligible effect on the outcome [14].

Anti-hypertensive Medications

**Figure 10:**
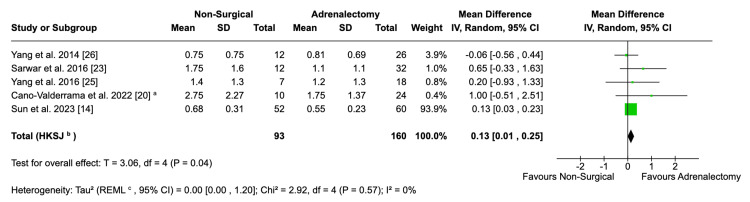
Forest plot visualising comparative number of anti-hypertensive medications taken by patients between surgical and non-surgical populations ^a ^Mean and standard deviation data from this study were converted from medians and ranges using the Luo-Wan method

Data pooled from five studies showed a small but significant difference between the number of anti-hypertensive medications taken by patients post-procedure (MD: 0.13; 95% CI: 0.01, 0.25; p = 0.04) Figure 10). This result was not affected by heterogeneity (I^2^ = 0%). A study by Sun et al. [14] reported this outcome with a median and range, so this was used to derive an estimated mean and SD.

Discussion

PA is thought to account for up to 13% of cases of hypertension, with diagnosis rates being as low as 1% [1,2,7]. Prior research has shown PA to be linked to a range of cardiovascular, renal and metabolic complications through mechanisms related to as well as independent of hypertension [1,27]. Patients with PA often present with preventable end-organ damage due to diagnostic delays. This delay is largely due to the lack of screening programmes and the non-specific presentation of PA [7,8]. The complications that occur as a result of these delays have a significant impact on the quality of life and increase the cost burden on healthcare systems. Furthermore, ever-increasing morbidity means that many patients may no longer be viable surgical candidates. Hence, more research is needed to determine whether non-surgical or minimally invasive options are suitable for poor surgical candidates or patients declining surgery.

PA was first brought to the medical community’s attention by Dr. Jerome Conn in 1955, from whom the syndrome gets its eponymous name, during the so-called 'golden age of endocrinology' [28]. The early mainstay of treatment was anti-hypertensive therapy followed by MRA. This was soon overtaken by surgical management with the widespread availability of laparoscopic surgery. While surgery was a highly successful and widely adopted mode of treatment, challenges with lateralisation techniques and an increasing pool of patients averse to or unfit for surgery necessitated further innovation. In 2004, the medical team of a 57-year-old gentleman in Jordan opted to manage his PA with RFA as they had the expertise available to do so [29]. They reported how the procedure was minimally invasive and was able to cure the patient’s hypertension, all while reducing his time spent in hospital and costs incurred. Since then, there has been keen interest in alternatives to surgery. RFA and catheter-based ablation have both shown promise [30,31]. FABULAS, a recent UK-based multi-centre study, showcased the immense promise of endoscopic-ultrasound guided thermal ablation of aldosterone-producing tumours identified via molecular imaging [1]. They showed the possibility of eliminating invasive procedures in both the lateralisation process and the ablation process. At the time of writing this paper, there is no mention of alternative procedures to LA in the treatment of PA in guidelines produced by the Endocrine Society or the National Institute for Health and CARE Excellence (NICE) [32,33]. 

This systematic review and meta-analysis aimed to evaluate the efficacy and safety of non-surgical treatments-namely ablation and embolisation-compared to LA for PA. While LA remains the gold standard for unilateral APAs, the emergence of minimally invasive techniques offers potential alternatives for patients who are unsuitable for surgery or decline operative intervention. Overall, this meta-analysis brought to light very promising findings. Ablation and embolisation are capable of producing similar rates of curing PA as surgical management while causing fewer procedure-related complications. Non-surgical management also shows promise in potentially reducing the length of operating time and length of hospital stays.

Our pooled analyses demonstrated that non-surgical approaches can achieve meaningful clinical and biochemical success rates as defined by the PASO criteria [5]. Surgical patients demonstrated a clinical success rate of 90.1%, whereas non-surgical patients achieved an 85.9% success rate. Similarly, biochemical success rates were 94.7% and 95.2% in the surgical and non-surgical groups, respectively. There was no heterogeneity among the pooled studies, and despite the marginally higher success rates for adrenalectomy, no statistical significance was observed between the two groups. These are highly promising results as these novel therapies are providing similar outcomes to LA, which has been taking place since 1992 [34].

Our analysis showed that the occurrence of complications was higher in surgical groups compared to non-surgical groups. Minor classifications were classed as Grade I or II according to the Clarien-Dindo classification [18]. The most common minor complications reported were pain, nausea and vomiting, and pyrexia, which all resolved with supportive care. The occurrence rate was 19.6% in the non-surgical groups compared to 23.6% in the surgical group, though this was statistically insignificant. Major complication data were not used in this meta-analysis as there were zero such events among patients undergoing ablation or embolisation. There were seven occurrences of major complications, as defined by the Clarien-Dindo system [18]. These included pancreatic duct damage, liver laceration, and vascular injury. These are well-known complications associated with surgery and contribute to increased morbidity and costs and negatively impact patient quality of life. The reduced incidence and severity of procedure-associated complications is a key factor in decision-making for both patients and doctors alike when evaluating the benefits and drawbacks of surgical management. 

Furthermore, given the nature of the procedures, non-surgical interventions had statistically significantly shorter operating times compared to adrenalectomy. This is because adrenalectomy generally requires careful dissection of the surrounding structures and vessels, and patients were under general anaesthesia (GA). Non-surgical techniques, however, did not require dissection of the tissues since electrodes were placed at the desired location under direct image guidance. Furthermore, many of the studies performed ablation under local anaesthesia, which requires less time than GA, shortening the total operating time. However, considerable heterogeneity was observed between the studies, which was not resolved by sensitivity analysis. This could be due to several factors. Firstly, the experience level of the surgeons and the use of different electrodes lead to discrepancies in procedure timing. Secondly, some studies involved embolisation, whereas others used ablation. Given that these two procedures are performed differently, the total operation time will vary. Moreover, some studies used CT guidance while others used ultrasound to locate the target tissues and the time taken for each imaging modality to be set up and used varies, favouring ultrasound. Finally, Sarwar et al. and Yang et al. opted for GA during their ablation procedures instead of local anaesthesia, which explains their considerably longer operating times compared to other studies [23,25]. The combination of these factors explains the heterogeneity observed, highlighting the need for future standardised studies to better determine operating times. This is vital as longer operating times are associated with a significantly higher risk of complications. This is often multi-factorial. Longer procedures mean longer time exposed to microbes; reduced efficacy of antibacterial and venous thromboembolism prophylaxis; tissue ischaemia and desiccation secondary to retraction; and more time for the risk of sterile field breaches.

The nature of the procedures further explains the discrepancy in the intraoperative blood loss. Laparoscopic procedures involved the dissection and ligation of the adrenal arteries and veins, which meant that blood loss was more likely in these patients. Data from the pooled studies reflect this in the forest plot, though it is statistically insignificant. There was considerable heterogeneity among the pooled studies, highlighting the need for further research with standardised techniques and measuring systems to better assess intraoperative blood loss. 

The use of GA, increased risk of complications, as well as increased risk of blood loss, explains why surgical patients had longer hospital stays compared to non-surgical patients. Adrenalectomy was performed under GA, and it is known that the effects of GA can last up to 24 hours [35]. Additionally, they lead to side effects such as nausea and vomiting, which would require a longer hospital stay for supportive management [36]. Nausea and vomiting were common minor complications reported, and given the fact that surgical patients were more likely to experience complications, it would explain their prolonged stay. The average length of stay was further increased for surgical patients due to the need for interventions for major surgical complications. The difference, however, is non-significant and considerable heterogeneity was observed. This heterogeneity can be explained by Sarwar et al. and Yang et al. opting for GA, which would have prolonged post-operative recovery and predisposed patients to complications such as nausea and vomiting, prolonging overall hospital stay [23,25]. Further research and training on non-surgical techniques will increase the confidence of clinicians in performing these procedures under local anaesthesia to allow for quicker recovery. 

Intraoperative hypertensive crises were defined as a systolic BP of more than 180 mmHg and a diastolic BP of greater than 120 mmHg. These were more commonly observed in the non-surgical group (9.4%) compared to the surgical group (2.9%). A prospective cohort study by Yamakado et al. observed a significant correlation between catecholamine levels and hypertensive crises during RFA of adrenal tumours [37]. The increase in catecholamines has been attributed to the release of the hormones during destruction of the tissue, electric stimulation by the ablation, as well as pain and anxiety related to the procedure [37]. Given the fact that the adrenal vein is not ligated as is done in adrenalectomy, the catecholamines are more likely to enter the systemic circulation and cause a hypertensive crisis [20]. These mechanisms explained the increased number of crises seen in our non-surgical group compared to the ablation group. However, the differences were not statistically significant, highlighting the need for more studies to accurately assess the safety of non-surgical techniques. To mitigate hypertensive crises, future studies should use close monitoring of patients and intraoperative blood pressure medication. Yamakado et al., in their study, found calcium channel blockers to be effective in controlling hypertensive crises but also recommended the use of alpha-adrenergic blockers given that the cause of the crisis is catecholamine release [37].

Our review found that patients undergoing non-surgical treatment had a higher mean number of antihypertensives post-procedure. This finding was statistically significant, and no heterogeneity was observed among the studies. This could be explained by the fact that previous research has shown that aldosterone production can be from both macronodules and micronodules [38]. Specimens were examined using immunochemistry to detect enzymes responsible for aldosterone production (CYP11B2), and they found these to be present in micronodules and clusters [38]. Compared to surgery, which removes the entire gland, non-surgical techniques only target macronodules, allowing aldosterone-producing micronodules to survive. The continued production of aldosterone will then lead to increased blood pressure and continued reliance on antihypertensives to control it. Despite this, non-surgical treatment remains a viable option as it achieves the clinical and biochemical targets as defined in the PASO criteria without the increased risk of surgical complications [5]. In order to address this discrepancy compared to LA, future protocols and training given to interventional radiologists should focus on identifying multiple sites for ablation. In the aforementioned FABULAS study, the number of areas ablated increased as the study progressed because the experience of the endoscopists increased, and they iterated their approach based on learnings from earlier cases [1].

The diagnosis and management of PA remains a challenge. The Endocrine Society guidelines suggest that the differentiation between bilateral adrenal hyperplasia and unilateral APAs should be done through AVS [32]. However, this remains a challenge as it is a difficult and underutilised skill in many parts of the world [39]. Previous retrospective studies have found that these techniques are not performed systematically, which was attributed to their reliance on imaging, the cost of the procedure, and the lack of expertise in performing AVS [39]. This, coupled with the novelty of using ablation for unilateral APAs, highlights the need for further studies to better assess ablation as an alternative to surgery. These studies must use a standardised screening and eligibility criteria, adopt a standardised approach for both the diagnosis and treatment of PA, and must ensure adequate follow-up to accurately assess clinical and biochemical outcomes as defined by the PASO criteria [5]. Several RCTs, such as the WAVE and PA-SELECT trials, are already underway to assess the efficacy of ablation and embolisation compared to LA, respectively. In addition, other retrospective and prospective studies are being conducted to evaluate these procedures. The results of these studies will shed more light on the efficacy and safety of these procedures and allow clinicians to more confidently explore alternatives to surgery with their patients. To help aid a change in management, more information needs to be gathered to determine the cost benefits, hospital expertise in performing these procedures, as well as assess the long-term impact on the patient's quality of life. 

## Conclusions

Minimally invasive techniques such as ablation and embolisation have the potential to serve as viable alternatives to surgery in treating PA. These techniques have shown comparable clinical and biochemical successes and fewer post-operative complications. Minimally invasive techniques did, however, show an increased risk of hypertensive crises and showed a higher reliance on blood pressure medications post-procedure compared to LA. Hence, treatment must be tailored to the patients' needs and preferences, and they must be given complete information on the outcomes of each procedure to make an informed choice. The authors keenly await the findings of RCTs and other studies currently being carried out, which will allow for more evidence to better assess the viability of minimally invasive techniques as alternatives to surgery.

## Appendices

**Table 3:** Database-specific search strategies used in this systematic review and meta-analysis

Database	Search strings
MEDLINE and Embase (via Ovid)	​​("primary aldosteronism" OR "primary hyperaldosteronism" OR "conn's" OR "aldosterone producing adenoma" OR "adrenal hyperplasia") AND ("radiofrequency ablation" OR "ultrasound ablation" OR "catheter ablation" OR "embolisation" OR "embolization") AND ("adrenalectomy" OR "adrenal surgery" OR "surgical management" OR "surgical removal" OR "surgical resection" OR "adrenal resection")
PubMed	​​("primary aldosteronism" OR "primary hyperaldosteronism" OR "conn's" OR "aldosterone producing adenoma" OR "adrenal hyperplasia") AND ("radiofrequency ablation" OR "ultrasound ablation" OR "catheter ablation" OR "embolisation" OR "embolization") AND ("adrenalectomy" OR "adrenal surgery" OR "surgical management" OR "surgical removal" OR "surgical resection" OR "adrenal resection")
Scopus	( "primary aldosteronism" OR "primary hyperaldosteronism" OR "conn's" OR "aldosterone producing adenoma" OR "adrenal hyperplasia" ) AND ( "radiofrequency ablation" OR "ultrasound ablation" OR "catheter ablation" OR "embolisation" OR "embolization" ) AND ( "adrenalectomy" OR "adrenal surgery" OR "surgical management" OR "surgical removal" OR "surgical resection" OR "adrenal resection" )
Web of Science	​​("primary aldosteronism" OR "primary hyperaldosteronism" OR "conn's" OR "aldosterone producing adenoma" OR "adrenal hyperplasia") AND ("radiofrequency ablation" OR "ultrasound ablation" OR "catheter ablation" OR "embolisation" OR "embolization") AND ("adrenalectomy" OR "adrenal surgery" OR "surgical management" OR "surgical removal" OR "surgical resection" OR "adrenal resection")
